# Bacterial gastroenteritis caused by the putative zoonotic pathogen *Campylobacter lanienae*: First reported case in Germany

**DOI:** 10.1099/acmi.0.000199

**Published:** 2021-01-25

**Authors:** Juliane Fornefett, Anne Busch, Sandra Döpping, Helmut Hotzel, Dagmar Rimek

**Affiliations:** ^1^​ Thuringian State Authority for Consumer Protection, Tennstedter Str. 8/9, 99947 Bad Langensalza, Thuringia, Germany; ^2^​ Friedrich-Loeffler-Institut, Institute of Bacterial Infections and Zoonoses, Naumburger Str. 96a, 07743 Jena, Thuringia, Germany

**Keywords:** butcher, *Campylobacter*, campylobacteriosis, enteritis, *lanienae*

## Abstract

Foodborne campylobacteriosis is the most common cause of human bacterial enteritis in Germany. *Campylobacter jejuni* and *Campylobacter coli* are the main causative agents for enteric disease, but a number of other species are involved, including rare ones. These rare *Campylobacter* spp. are emerging zoonotic pathogens in humans due to increasing international movement of supplies, livestock and people. *Campylobacter lanienae* was first isolated from healthy abattoir workers in Switzerland and at first its pathogenic potential for humans was considered to be low. Recently, the first case of *Campylobacter lanienae *-associated human enteritis was reported in Canada. Here, we describe a case of mild *Campylobacter lanienae *-associated enteritis with subsequent asymptomatic excretion in a butcher. The isolate is available at the TLV strain collection (no. TP00333/18). This first reported case of human *Campylobacter lanienae* campylobacteriosis in Germany demonstrates the agent’s likely zoonotic pathogenicity.

## Introduction

*Campylobacter (C.)* species are the most frequent cause of human bacterial enteritis in Germany [1]. Transmission to humans is mainly food-associated [1]; 50–90 % of human infections are related to the consumption of poultry meat [1]. Among the approximately 30 species, *Campylobacter jejuni* and *Campylobacter coli* are the most common causative agents of campylobacteriosis in humans [1]. However, rare and emerging *Campylobacter* species should not be underestimated and have to be monitored.

*Campylobacter lanienae* appears to be a natural inhabitant of the gastrointestinal tract of healthy farm and feral animals, such as pigs, ruminants, poultry and probably rodents [2–11]. It was initially considered to be a non-pathogenic commensal in humans when it was isolated from stool samples of healthy abattoir workers in Switzerland in 2000 [12]. However, in 2016 the first case of *Campylobacter* enteritis due to *C. lanienae* was observed in Canada [13]. The patient showed diarrhoea, lower abdominal cramps, nausea, vomiting and low-grade fever.

Here, we report the first case of mild *C. lanienae-*associated enteritis in Germany and describe the molecular biological investigation of isolate TP00333/18.

## Case presentation

In January 2018, a male in his fifties presented with sudden onset of nausea and diarrhoea to his physician in central Germany. He was in a good physical condition and reported no other symptoms. A stool sample was sent to a private laboratory and a *Campylobacter* species was isolated without further identification. Specific treatment was not prescribed and the mild symptoms resolved completely after <3 days. The case was notified to the local Public Health Department according to the German Infection Protection Act (IfSG) 13 days after the onset of symptoms.

The patient reported no history of underlying intestinal disease, previous antimicrobial therapy or recent travel. He worked as an employed butcher. The company, a meat-processing plant, did not slaughter any live animals but processed pig carcasses. These were provided in pre-cut pieces by different international vendors. The patient lived in a rural area in a house together with one other family member, where they kept canary birds, as well as chickens and rabbits for consumption. Neither the family member nor the co-workers at the meat factory reported any symptoms of enteritis.

The public health authority imposed a temporary work ban according to the IfSG and transferred a fresh follow-up stool sample from the patient and his family member to the Thuringian State Authority for Consumer Protection for bacterial investigation. Colonies grown from the patient’s soft stool sample on *Campylobacter* agar had a smooth, brightly coloured, grey–brown appearance with irregular edges and were non-swarming. Identification of the isolate TP00333/18 revealed the rare species *C. lanienae *. Antimicrobial susceptibility testing showed no resistance against antimicrobial agents commonly recommended for treatment, such as macrolides, fluoroquinolones and tetracyclines.

The work ban was maintained in agreement with the Public Health Department and further follow-up stool samples from the patient and his family member were submitted at 3-day intervals. In total, *C. lanienae* was isolated from eight consecutive stool samples from the patient, covering a period of 40 days. Subsequently, four negative results were obtained and the work ban was lifted. A total of three samples from his asymptomatic family member gave negative results.

Since this was a singular case of campylobacteriosis, the Public Health Department neither investigated the source of transmission nor examined stool samples from the employees of the meat processing plant.

## Investigation

### Methods

#### Cultivation of bacteria

Stool samples were plated on Blood-Free *Campylobacter* agar (Karmali, Oxoid Deutschland GmbH, Wesel, Germany) and incubated at 42 °C for 48 h under microaerophilic conditions (Anoxomat Advanced Instruments, Norwood, MA, USA; 5.9 % O_2_, 3.6 % CO_2_, 7.2 % H_2_, 83.3 % N_2_).

To exclude other enteric bacterial pathogens, stool samples were subjected to other standard culture procedures. For the detection of *Salmonella enterica* and *Shigella* spp., Desoxycholate Citrate Agar (DCA) and McConkey Agar (both Oxoid Deutschland GmbH) were incubated aerobically at 37 °C for 48 h. For the enrichment of *Salmonella* spp., Selenite Broth (Oxoid Deutschland GmbH) was incubated aerobically at 37 °C for 24 h and subcultivated on DCA (incubated as described above). *Yersinia* sp. detection was performed using *Yersinia* Selective Agar (CIN, Oxoid Deutschland GmbH), which was incubated aerobically at 25 °C for 48 h. To identify toxigenic *Escherichia coli *, the stool sample was plated on Sorbitol McConkey Agar (SMAC) and suspended in Tryptone Soy Broth (TSB) with 10 mg l^−1^ novobiocin (both Oxoid Deutschland GmbH). *E. coli* media were incubated as described above for *Salmonella* for 24 h. PCR for toxin gene detection and EIA for the detection of toxin production were conducted using the RIDAGENE *E. coli* Stool Panel I kit from the mixed culture on SMAC and the RIDASCREEN Verotoxin Enzyme Immunoassay (both R-Biopharm AG, Darmstadt, Germany) from the overnight TSB culture, respectively.

#### Preliminary identification

Colonies on Karmali agar suspected to be *Campylobacter* spp. were tested for the presence of catalase (10 % hydrogen peroxide, Merck KGaA, Darmstadt, Germany) and cytochrome oxidase (Bactident oxidase, Merck KGaA). Catalase- and oxidase-positive colonies were used for further identification.

#### Matrix-assisted laser desorption/ionization time-of-flight mass spectrometry (MALDI-TOF MS)

MALDI-TOF MS was used for species identification. Spectra were generated using a Microflex LT instrument and analysis was performed using Biotyper 3.1 software (both Bruker Daltonik GmbH, Bremen, Germany).

#### Antimicrobial susceptibility testing

Antimicrobial susceptibility testing was performed for streptomycin (25 µg), penicillin (10 µg), ciprofloxacin (5 µg), tetracycline (30 µg), gentamicin (10 µg), amoxicillin/clavulanic acid (3 µg) and erythromycin (30 µg) (all Oxoid Ltd, Basingstoke, UK) using disc diffusion tests according to European Committee on Antimicrobial Susceptibility Testing (EUCAST) [14] and Clinical and Laboratory Standards Institute (CLSI) [15] guidelines.

### Partial sequencing of 16S rRNA and species-specific PCR

Partial sequencing of the 16S rRNA gene was performed using the ABI Prism 3500 Genetic Analyzer (Applied Biosystems, Foster City, CA, USA) after purification of bacterial DNA using the QIAamp DNA Mini Kit (Qiagen GmbH, Hilden, Germany) from an overnight culture on Columbia Sheep Blood Agar (Oxoid Deutschland GmbH). PCR amplification was performed using primer pair 16S-FW/16S-Rev (5′-GAA GAG TTT GAT CAT GGC TCA G-3′/ 5′-ACG ACA GCC ATG CAG CAC CT-3′) and thermal cycling with initial denaturation at 95 °C for 10 min, followed by 35 cycles of denaturation at 95 °C for 60 s, annealing at 53 °C for 60 s and an extension at 72 °C for 90 s, with a final extension at 72 °C for 10 min. The sequence was identified using the National Center for Biotechnology Information (NCBI) blastn database (version 2.8.0).

In addition, species-specific PCR was performed. After overnight cultivation of isolate TP00333/18 on Mueller–Hinton Agar (Oxoid Deutschland GmbH), DNA was extracted using the High Pure PCR Template Purification Kit (Roche Diagnostics, Mannheim, Germany). PCR was conducted using primers CLAN76F [12] and CLANL521021R [16]. Cycling consisted of initial denaturation at 96 °C for 60 s, 35 cycles of denaturation (96 °C for 15 s), annealing (53 °C for 60 s) and an extension (72 °C for 60 s). PCR resulted in a 920 bp amplicon.

#### Multilocus sequence typing (MLST)

MLST was conducted using amplification and sequencing primers under the conditions described by Miller *et al*. [17]. DNA sequencing of purified PCR products was carried out by cycle sequencing using the BigDye Terminator Cycle Sequencing Ready Reaction Kit (Applied Biosystems). Nucleotide sequences were determined on an ABI Prism 3130 Genetic Analyzer (Applied Biosystems). The sequence type was determined using the *Campylobacter* MLST Home Page (http://pubmlst.org/campylobacter/).

#### Whole-genome sequencing (WGS)

DNA for WGS was prepared from a 2 ml culture in Mueller–Hinton Broth (Oxoid Deutschland GmbH) after incubation at 37 °C for 48 h under microaerophilic conditions, using the DNA extraction kit as described above. The sequencing library was generated using the Nextera XT DNA Library Prep Kit (Illumina, Inc., San Diego, CA, USA). From an Illumina MiSeq run with an average read length of 300 bp and an expected insert size of 350 bp, for the MLST amplicons 50,000 paired-end reads and for the whole genome, 150,000 paired-end reads were generated (mean sequencing depth of >10 reads with a standard deviation of 6 reads for the whole-genome assembly, mean sequencing depth of >1,000 with a standard deviation of 68 reads). Further processing of the WGS data included quality trimming and assembly [included in SPAdes version 3.12.1 in Bayes Hammer mode (--careful)] [18]. Annotation was performed with Prokka [19]. Additionally, PhyloPhlAn [20] was used to assign microbial phylogeny, which was visualized with Dendroscope [21].

## Results

### Isolation and identification

The bacterial isolate TP00333/18 was isolated from a stool sample on Karmali agar under microaerophilic conditions at 42 °C after 2 days. Using MALDI-TOF MS, it was identified as *C. lanienae *. Because of the low score of 1.8 from MALDI-TOF MS identification, partial sequencing of the 16S rRNA gene was carried out, as well as species-specific PCR. Both methods confirmed the isolate as *C. lanienae *. Comparison of the 16S rRNA amplicon with the blastn database (version 2.8.0) revealed a 99–100 % sequence identity with the *C. lanienae* isolate G718b 16S rRNA gene (accession number AY165045.1).

The isolate was resistant to penicillin and amoxicillin/clavulanic acid. It was susceptible to streptomycin, ciprofloxacin, tetracycline, gentamicin and erythromycin. Other enteric pathogens such as *Salmonella *, *Yersinia, Shigella* or *E. coli* were not isolated.

### MLST and WGS

**Fig. 1.**
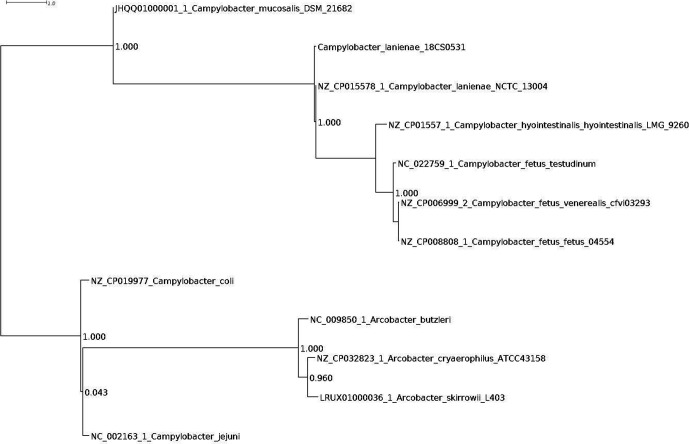
PhyloPhlAn was used to assign microbial phylogeny as described previously [20] and this was visualized with Dendroscope [21]. 18CS0531 corresponds to *C. lanienae* isolate TP00333/18.

The assembly of the WGS of the isolate gave low coverage, and the isolate thus had a lower estimated genome size than the reference genome of *C. lanienae* NCTC13004 (NZ_CP015578.1). The genome assembly was represented by 886 contigs, with the largest contig consisting of 14,522 bases, giving a total estimated length of 1,342, 700 bases and an N_50_ contig length of 1,793 bases. Annotation features include 1,252 genes and 2 rRNAs, 35 tRNAs and 1 transfer-messenger RNA (tmRNA) for a total sequence length of 1,413, 167 bases. The GC content of the draft genome sequence was 37.5 %. Nevertheless, a correct taxonomic differentiation of the isolate TP00333/18 from other pathogenic *Campylobacter* species was possible with PhyloPhlAn, as shown in Fig. 1. MLST of TP00333/18 resulted in the alleles given in Table 1. It corresponded to an unknown sequence type.

**Table 1.** Results of MLST of *C. lanienae* isolate TP00333/18

Locus	Closest match to allele	Differences to known allele
*asp*A	21	–
*atp*A	2	–
*gln*A	7	2
*glt*A	39	5
*gly*A	68	1
*pgm*	2	5
*tkt*	3	7

## Discussion

It appears reasonable to assume that the causative agent for the mild gastroenteritis was indeed *C. lanienae,* as both our laboratory and the private laboratory that examined the first stool sample detected *Campylobacter* sp.

*C. lanienae* was first described in 2000 in Switzerland [12]. During a routine hygiene screen of asymptomatic abattoir workers, a *Campylobacter *-like organism was isolated from faecal samples from two employees. Phenotypic and molecular characterization revealed a new species that was phylogenetically most related to *Campylobacter hyointestinalis* subsp. *hyointestinalis *, *Campylobacter fetus* and *Campylobacter mucosalis* [12]. In 2003, a study determined that pigs were the main hosts of *C. lanienae *, as it was only isolated from pig faeces and not faeces from cattle or broilers [3]. Two studies in the same year succeeded in detecting *C. lanienae* in bovine faeces and confirmed cattle as another main host [2, 16]. Genetic examination revealed two different genetic clusters, according to the respective animal reservoir [4]. In the following years, *C. lanienae* was also detected in sheep [6], wild boars [7–9], feral ruminants [8, 9], chickens [10] and laboratory chinchillas [11], indicating a broad host spectrum similar to that of other *Campylobacter* species. Recently, the first case of human enteritis caused by *C. lanienae* was reported from Canada [13]. The female patient was hospitalized with a 5-day history of diarrhoea, lower abdominal cramps, nausea, one episode of bilious vomiting and low-grade fever. She had 10 or more diarrhoeic stools per day as well as during the night, and had noticed blood mixed with the stools. Questioning revealed a history of irritable bowel syndrome 1 year before admission. The patient’s symptoms resolved without treatment after 9 days. In contrast, our patient showed only mild symptoms of sudden nausea and diarrhoea that were self-limiting. No predisposing gastrointestinal diseases or any previous antimicrobial treatment was recorded.

Based on the reports from different countries of Europe (including Germany), North America and Asia [2–10, 16, 22], it is reasonable to assume the worldwide presence of this organism in animals. In our case, the patient regularly came into close contact with putatively contaminated pig carcasses as well as animals from his private husbandry. Unfortunately, meat or meat products from the plant, as possible sources of the infection, were not investigated, as this was only a single case of mild gastroenteritis with no direct suspicion of a foodborne source and/or an outbreak situation. Moreover, the first notification reached the Public Health Department very late after the actual event, which limited the chances of a successful investigation. The Public Health Department also declined to perform an examination of the co-workers in the meat plant. The German Infection Protection Act (IfSG) does not require a follow-up for asymptomatic contact persons in the case of *Campylobacter* infection [23]. For the same reason, the patient’s animal husbandry was not examined. We consider the pig carcasses at the plant to be a more likely cause of the infection, although clear evidence for this is lacking. Both the patient’s unaffected family member living in the same household and this being a singular event support the hypothesis of a singular exposure in the factory rather than a permanent animal exposure in the private setting. On the other hand, none of the co-workers showed any signs of enteritis.

The prevalence of *C. lanienae* in animals and of exposed humans should be subjects of further investigations, especially as *C. lanienae* can be considered to be a potential cause of human gastroenteritis [13] and some isolates of animal origin showed substantial rates of antimicrobial resistance [5, 9]. Nevertheless, antimicrobial resistance in *C. lanienae* isolates of wildlife origin is still uncommon, in contrast to resistance encountered among frequently isolated human pathogenic species such as *Campylobacter coli* [8]. To the best of our knowledge, the resistance situation of *C. lanienae* isolates from farm animals has not been studied, yet. In the present case, the isolate was fully susceptible to streptomycin, ciprofloxacin, tetracycline, gentamicin and erythromycin.

Species identification of *Campylobacter* isolates should always be performed for newly identified cases. Although we used sequencing and species-specific PCR, accurate identification to species level is possible by MALDI-TOF MS. This is a rapid method that is available in most microbiology laboratories today.

## Conclusions

Thermophilic *Campylobacter* species are a common cause of foodborne gastroenteritis in Germany. Frequently detected species are *C. jejuni* and *C. coli *, but rare species like *C. lanienae* are increasingly being detected, especially within the context of international movement of supplies, livestock and people. We report the first case of gastroenteritis in Germany caused by the putative zoonotic pathogen *C. lanienae* in a butcher who had come into regular contact with pig carcasses that were supplied by international vendors. Routine laboratory detection that focuses on common species may miss rare and emerging species, and such species should also be captured by surveillance efforts.
